# Reversible Chemogenetic Fluorescence Labeling with pFAST in *C. elegans*

**DOI:** 10.17912/micropub.biology.001980

**Published:** 2026-01-10

**Authors:** Zixu Wang, Dominique Langevin, Vivian Chen, Jihong Bai

**Affiliations:** 1 Basic Sciences Division, Fred Hutchinson Cancer Center, Seattle, Washington, USA; 2 University of California, Davis, California, USA; 3 Fred Hutchinson Cancer Center Summer Undergraduate Research Program, Fred Hutchinson Cancer Center, Seattle, Washington, USA; 4 University of Puget Sound, Tacoma, Washington, USA; 5 Lakeside School, Seattle, Washington, USA

## Abstract

The promiscuous fluorescence-activating and absorption-shifting tag (pFAST) enables reversible chemogenetic labeling with multiple fluorogens. We generated a single-copy tandem pFAST (td-pFAST) transgenic *Caenorhabditis elegans* strain expressed in pharyngeal muscle. In dissected worms, lime fluorogen produced rapid fluorescence that was efficiently quenched by the competing ligand darth, demonstrating reversibility. Amber and coral fluorogens also produced reversible signals with distinct emission spectra, supporting multicolor labeling. However, fluorogen delivery by soaking intact worms failed, indicating cuticle permeability remains a barrier. These findings establish td-pFAST as a functional probe for reversible, multicolor labeling in dissected *C. elegans* tissues.

**Figure 1. Reversible td-pFAST labeling of the transgenic C. elegans pharynx:**
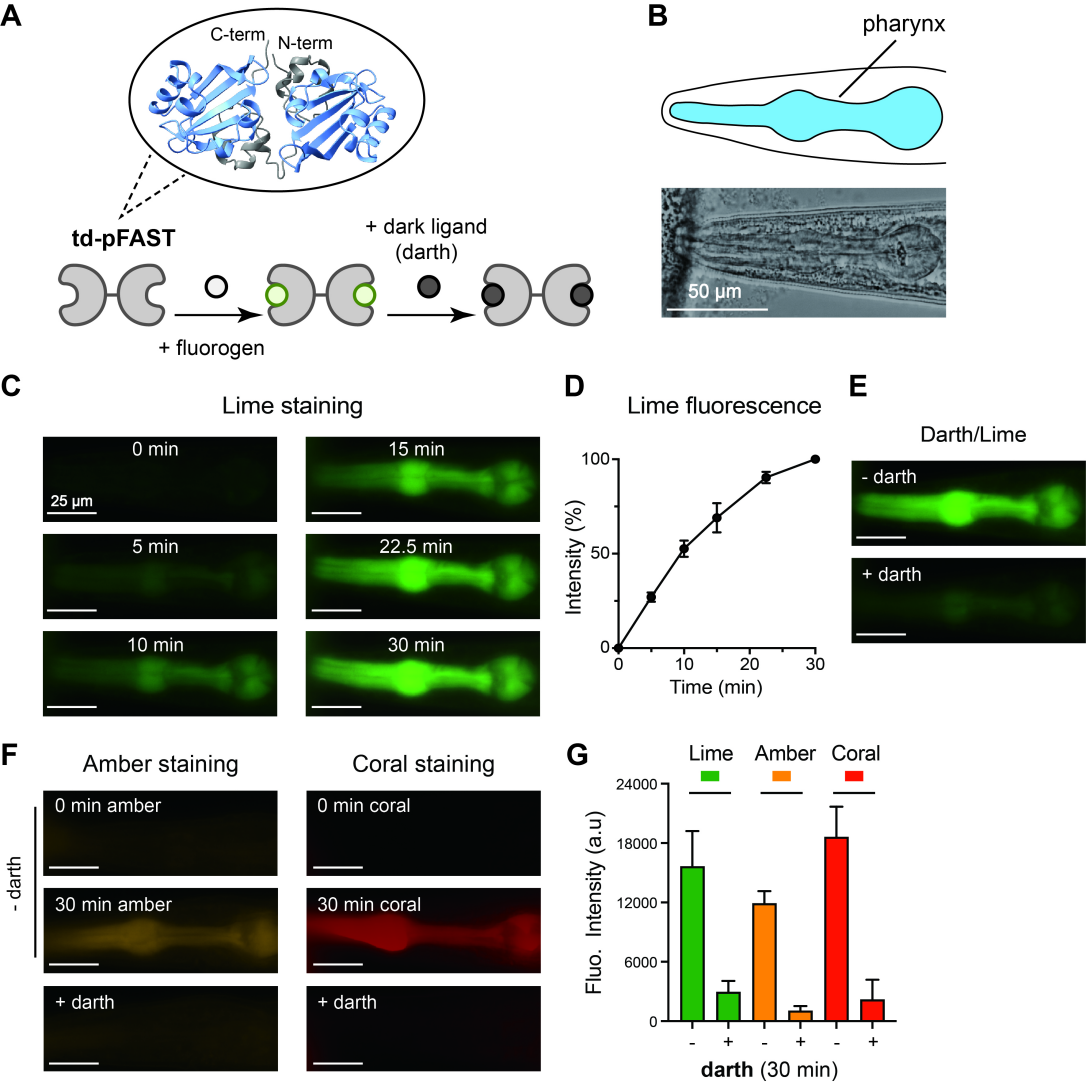
(A) Schematic of the AlphaFold-predicted structure of the tandem dimer pFAST protein (td-pFAST; *top* ) and fluorogen binding and quenching by darth ( *bottom* ). (B) Schematic ( *upper* , pharynx shown in blue) and corresponding bright-field image ( *lower* ) of the *C. elegans* head. Scale bar = 50 μm. (C) Lime fluorescence in dissected pFAST worms at 0, 5, 10, 15, 22.5, and 30 min of incubation. Scale bar = 25 μm. The td-pFAST coding sequence was codon-optimized for *C. elegans* , placed under the *myo-2* promoter ( *myo-2 p* ), and inserted at the *ttTi5605* locus on chromosome II using Mos1-mediated single-copy insertion. (D) Quantification of lime fluorescence over time in pFAST transgenic worms. Fluorescence intensity was normalized within each worm such that the 0- and 30-minute time points correspond to 0% and 100%, respectively. Mean ± SEM are shown. n = 6. (E) Darth quenching of lime fluorescence. After 30 min of incubation with lime, worms were washed twice with extracellular solution to remove excess lime (- darth), followed by addition of darth to quench fluorescence (+ darth; image shown at 30 min after darth addition). Scale bar = 25 μm. (F) Representative images of amber and coral labeling at 30 minutes and darth quenching (+ darth; 30 min after darth addition). Scale bar = 25 μm. (G) Quantification of darth quenching (+ darth; at 30 min after darth addition) of lime (n = 4), amber (n = 6), and coral (n = 5) fluorescence (mean ± SEM).

## Description

Genetically encoded fluorescent proteins such as GFP and RFP provide robust labeling for cell biology studies but are limited by irreversibility and by some restrictions of multiplexing due to spectral overlap (Chalfie 2009, Tsien 2009). The fluorescence-activating and absorption-shifting tag (FAST) offers an extension to the toolkit of fluorescent tags: it fluoresces only when bound to specific fluorogenic ligands, and fluorescence can be reversed by removing or exchanging the ligand (Benaissa et al 2021, Bottone et al 2025, Li et al 2017, Plamont et al 2016, Tebo et al 2021, Tebo et al 2018). A promiscuous variant, pFAST, binds multiple fluorogens across the visible spectrum (Benaissa et al 2021). While pFAST has been applied in cultured cells, its performance in multicellular organisms is unclear. We tested whether tandem pFAST (td-pFAST) could provide reversible and multicolor labeling in *C. elegans* .

We constructed a td-pFAST reporter using a tandem design to increase signal intensity ( Figure 1A ). The codon-optimized td-pFAST coding sequence was placed under the *myo-2* promoter to drive expression in pharyngeal muscle. The pharynx was selected because it is a large and autofluorescence-free tissue that can be easily identified ( Figure 1B ). Transgenic animals were generated by Mos1-mediated single-copy insertion at the *ttTi5605* site on chromosome II (Frokjaer-Jensen et al 2012, Frokjaer-Jensen et al 2008), verified by PCR amplification and DNA sequencing.

In dissected worm preparations, lime fluorogen (HMBR) produced detectable fluorescence in pharynx within 5 minutes, reaching approximately 50% of maximal intensity by 10 minutes and continuing to increase over 30 minutes ( Figure 1C- D). When the non-fluorescent competing ligand darth (HBIR-3M) was applied after lime labeling, pharyngeal fluorescence decreased substantially, with more than 80% of the signal quenched within 30 minutes ( Figure 1E and 1G). These measurements demonstrate efficient cellular permeability, fluorescence activation, and robust reversibility of td-pFAST labeling in dissected tissue.

We next tested two additional fluorogens, amber (HBR-3,5DM) and coral (HBR-3,5DOM), in dissected preparations. Both produced robust fluorescence with distinct emission spectra. Labeling kinetics were similar to lime, with steady signal accumulation over 30 minutes ( Figure 1F ). Following ligand exchange with darth, fluorescence from both amber and coral was efficiently quenched to extents comparable to lime, demonstrating reversible labeling across all three fluorogens ( Figure 1F- 1G). This ligand flexibility enables labeling of the same tissue in different spectral channels.

Finally, we attempted fluorogen labeling in intact animals by soaking worms in solutions containing lime, amber, or coral. No detectable fluorescence was observed in the pharynx at either 30 minutes or 24 hours after incubation. The *C. elegans* cuticle restricts entry of small molecules, likely limiting fluorogen access to internal tissues. Thus, this inability to label intact worms indicates that current applications of td-pFAST are best suited to dissected preparations.

Several factors should be considered for future improvement. First, better fluorogen design to enhance cuticle penetration, co-treatment with permeabilizing agents, or direct delivery methods such as microinjection could extend utility to live, intact animals. Another possible approach would be to use worms with impaired cuticle integrity, which could improve fluorogen access to internal tissues. However, defects in cuticle structure can alter physiology, stress responses, and overall animal health, potentially complicating interpretation of fluorescence signals.

Second, our study tested fluorogen concentrations of 10 µM for both short and extended incubations. Reported binding affinities of FAST and pFAST fluorogens are typically in the submicromolar range (Benaissa et al 2021), suggesting that the concentrations used here are already well above the dissociation constants. This indicates that limited uptake, rather than insufficient fluorogen concentration, is likely the primary barrier in intact worms. While higher concentrations could be explored, careful titration would be required to assess whether increased dosing improves labeling without introducing nonspecific background or toxicity.

In addition, pFAST excitation and emission spectra do not perfectly align with traditional fluorophores like GFP or mCherry. Suboptimal filter sets can reduce apparent brightness and contribute to variability between fluorogens. Further tuning of fluorogen spectra to better match commonly used filter sets, together with optimized optics, may improve detection sensitivity.

In summary, td pFAST provides reversible, multicolor labeling in dissected *C. elegans* tissues, offering temporal control and spectral flexibility beyond conventional fluorescent proteins. While current applications are confined to dissected preparations due to cuticle permeability barriers, the system demonstrates the potential of chemogenetic probes to expand experimental flexibility in *C. elegans* .

## Methods

***C. elegans* Culture -** Worm strains were maintained at 22°C on NGM (nematode growth medium) agar plates seeded with *Escherichia coli* OP50 bacteria according to standard protocols. Unless otherwise specified, all experiments were carried out using day 1 adult hermaphrodites.

**Molecular Biology -** DNA plasmids were assembled using the Gibson assembly method (Gibson et al 2009), and all constructs were sequence-verified. The coding sequence for tandem pFAST (td-pFAST) was codon-optimized for *C. elegans* expression and synthesized by Twist Bioscience, Inc. Promoters included *myo-2 p* for pharyngeal expression and *rps-0p* for pan-tissue expression. The plasmid BJP-F466 was used for microinjection to generate single-copy transgenes carrying *myo-2 p::td-pFAST:: tbb-2 3′UTR* with *rps-0p::hygR:: let-858 3′UTR* as the selection marker.

*pFAST amino acid sequence:*

MAIAEHVAFGSEDIENTLANMDDEQLDRLAFGVIQLDGDGNILLYNAAEGDITGRDPKQVIGKNFFKDVAPGTDTPEFYGKFKEGAASGNLNTMFEWTIPTSRGPTKVKVHLKKALSGDRYWVFVKRVSAGGSAAAEHVAFGSEDIENTLANMDDEQLDRLAFGVIQLDGDGNILLYNAAEGDITGRDPKQVIGKNFFKDVAPGTDTPEFYGKFKEGAASGNLNTMFEWTIPTSRGPTKVKVHLKKALSGDRYWVFVKRVAAA*

*pFAST DNA sequence:*

ATGGCGATCGCAGAGCATGTCGCCTTCGGCAGTGAGGATATCGAAAATACTCTCGCAAATATGGACGATGAACAGCTCGACAGACTCGCATTCGGAGTTATTCAGCTCGATGGAGATGGTAATATTTTGTTGTACAACGCTGCCGAAGGAGATATCACTGGACGTGATCCGAAGCAGGTGATAGGTAAAAACTTCTTCAAGgtaagtttaaacatatatatactaactaaccctgattatttaaattttcagGATGTCGCTCCAGGAACTGACACTCCGGAGTTTTACGGAAAATTTAAGGAGGGTGCAGCAAGTGGAAATCTGAATACCATGTTTGAGTGGACCATACCAACTAGTCGAGGACCAACAAAGGTCAAAGTTCATTTGAAAAAAGCTTTGTCCGGAGATCGTTACTGGGTGTTCGTAAAGCGAGTTTCGGCAGGCGGATCCGCGGCCGCTGAACACGTAGCTTTCGGTAGTGAAGATATCGAGAACACCCTTGCGAACATGGATGACGAACAGTTAGATCGTCTCGCATTCGGAGTAATTCAATTGGATGGTGACGGAAATATTCTTCTTTATAACGCCGCAGAAGGTGATATCACCGGACGAGACCCGAAGCAAGTTATTGGCAAAAATTTTTTCAAAGACGTTGCGCCAGGCACTGACACCCCTGAATTCTACGGTAAATTTAAAGAAGGAGCAGCATCGGGCAATCTTAATACAATGTTCGAGTGGACAATTCCTACCTCTAGAGGTCCTACAAAAGTGAAAGTGCACCTCAAAAAAGCATTATCAGGAGACCGATACTGGGTCTTTGTCAAACGTGTTGCGGCCGCATAA

**Transgenes and Germline Transformation -** Single-copy transgenic animals were produced using Mos1-mediated insertion via microinjection (Frokjaer-Jensen et al 2012) into BJH2002 worms carrying the *ttTi5605* Mos1 site on chromosome II. Hygromycin B (400 µl of 5 mg/ml) was applied to plates two days post-injection to select for animals with transgene insertions. The resulting strain, BJH3570 ( *pekSi726* ), carrying *myo-2 p::td-pFAST:: tbb-2 3′UTR* and *rps-0p::hygR:: let-858 3′UTR* , was verified by PCR amplification and DNA sequencing.

**pFAST Staining with Fluorogenic Ligands -** Fluorogenic ligands were obtained from InvivoChem or TwinkleFactory (https://www.the-twinkle-factory.com). HMBR (lime) was purchased from InvivoChem. HBR-3,5DM (amber), HBR-3,5DOM (coral), and HBIR-3M (darth) were purchased from TwinkleFactory. Ligands were dissolved in DMSO and diluted to 10 µM working stocks in M9 buffer.

For staining dissected worms, day-1 young adult animals were immobilized on Sylgard-coated coverslips using Histoacryl Blue glue (B. Braun Group) and dissected in extracellular solution with a sharp glass pipette (Zhang et al 2022). Dissected worms were further paralyzed with 30 mM sodium azide. Dissected samples were incubated with 5 µM fluorogenic ligands. Fluorescence images were collected at 5, 10, 15, 22.5, and 30 minutes. After 30 minutes, ligands were washed out with extracellular solution, followed by the addition of darth (1.25 µM) for competitive replacement. Imaging was collected again at 5, 10, 15, 22.5, and 30 minutes post-addition of darth. For intact animals, worms were incubated in 20 µl M9 buffer containing 10 µM fluorogenic ligands, and fluorescence was assessed at 30 minutes and 24 hours.

**Fluorescence Imaging -** Fluorescence imaging was performed using an inverted Leica DMi8 microscope equipped with a 40x water immersion objective (1.10 NA), a Lumencor SPECTRA X Light Engine, and an Andor iXon Life 888 EMCCD camera. Lime fluorescence was collected using the green fluorescent protein filter set. Coral fluorescence was captured using the mCherry filter set. Amber fluorescence was collected using the SpX-Q filter set. Images were processed with the FIJI package of ImageJ. Background fluorescence was determined per sample from regions adjacent to the pharynx and subtracted from pharyngeal values. Fluorescence intensity was normalized to the 30-minute value, reported as 100%.

**Quantification and Statistical Analysis -** Fluorescence intensity measurements were quantified using ImageJ. Data are presented as means ± standard error. Statistical analyses were performed using GraphPad Prism 10. Comparisons between two groups were assessed by two-tailed Student's t test. Multiple group comparisons were analyzed by one-way ANOVA followed by Dunnett's post hoc test. P values < 0.05 were considered statistically significant.
